# Emergence of Multidrug-Resistant *Salmonella enterica* Subspecies *enterica* Serovar Infantis of Multilocus Sequence Type 2283 in German Broiler Farms

**DOI:** 10.3389/fmicb.2020.01741

**Published:** 2020-07-17

**Authors:** Silvia García-Soto, Mostafa Y. Abdel-Glil, Herbert Tomaso, Jörg Linde, Ulrich Methner

**Affiliations:** Institute of Bacterial Infections and Zoonoses, Friedrich-Loeffler-Institute, Jena, Germany

**Keywords:** *Salmonella* Infantis, broiler, emergence, multidrug resistance, pESI-like plasmid, whole genome sequencing

## Abstract

During the last decade, *Salmonella enterica* subspecies *enterica* serovar Infantis (*S*. Infantis) has become more prevalent across Europe with an increased capability to persist in broiler farms. In this study, we aimed to identify potential genetic causes for the increased emergence and longer persistence of *S*. Infantis in German poultry farms by high-throughput-sequencing. Broiler derived *S*. Infantis strains from two decades, the 1990s (*n* = 12) and the 2010s (*n* = 18), were examined phenotypically and genotypically to detect potential differences responsible for increased prevalence and persistence. *S*. Infantis organisms were characterized by serotyping and determining antimicrobial susceptibility using the microdilution method. Genotypic characteristics were analyzed by whole genome sequencing (WGS) to detect antimicrobial resistance and virulence genes as well as plasmids. To detect possible clonal relatedness within *S*. Infantis organisms, 17 accessible genomes from previous studies about emergent *S*. Infantis were downloaded and analyzed using complete genome sequence of SI119944 from Israel as reference. In contrast to the broiler derived antibiotic-sensitive *S*. Infantis strains from the 1990s, the majority of strains from the 2010s (15 out of 18) revealed a multidrug-resistance (MDR) phenotype that encodes for at least three antimicrobials families: aminoglycosides [*ant(3“)-Ia*], sulfonamides (*sul1*), and tetracyclines [*tet(A)*]. Moreover, these MDR strains carry a virulence gene pattern missing in strains from the 1990s. It includes genes encoding for fimbriae clusters, the yersiniabactin siderophore, mercury and disinfectants resistance and toxin/antitoxin complexes. In depth genomic analysis confirmed that the 15 MDR strains from the 2010s carry a pESI-like megaplasmid with resistance and virulence gene patterns detected in the emerged *S*. Infantis strain SI119944 from Israel and clones inside and outside Europe. Genotyping analysis revealed two sequence types (STs) among the resistant strains from the 2010s, ST2283 (*n* = 13) and ST32 (*n* = 2). The sensitive strains from the 1990s, belong to sequence type ST32 (*n* = 10) and ST1032 (*n* = 2). Therefore, this study confirms the emergence of a MDR *S*. Infantis pESI-like clone of ST2283 in German broiler farms with presumably high tendency of dissemination. Further studies on the epidemiology and control of *S*. Infantis in broilers are needed to prevent the transfer from poultry into the human food chain.

## Introduction

*Salmonella enterica* subspecies *enterica* serovar Infantis (*S*. Infantis) belongs to the group of *Salmonella* serovars, which plays a major epidemiological role in humans and animals. In the European Union (EU), *S*. Infantis has been the third most common serovar in humans since 2006 with a relative share between 1% and 2% (EFSA, 2019). Although this serovar is prevalent also in pigs and cattle (Rajic et al., 2005; Lindqvist and Pelkonen, 2007), poultry especially broiler and their products have been identified as one of the most important sources of human infection with *S*. Infantis (EFSA, 2019, 2020). In 2018, *S*. Infantis was the most frequently reported serovar in fowl in the EU (EFSA, 2019), accounting for 36.7% of all *Salmonella* isolates. Moreover, unlike previous years, *S*. Infantis was not only detected in a few numbers of countries but widespread among most member states and massively reported from broilers (36.5% of all serotyped isolates) and broiler meat (56.7%).

During the last years, antimicrobial resistance has emerged in *S*. Infantis organisms from different animal sources and humans in various European countries (Nógrády et al., 2012; EFSA, 2020). Increasing incidence and dissemination of different multidrug-resistant (MDR) *S*. Infantis clones in broiler populations resulted in spreading of the organisms in the food chain and via poultry products to humans in countries such as Hungary (Olasz et al., 2015), Italy (Franco et al., 2015), Switzerland (Hindermann et al., 2017), Slovenia (Pate et al., 2019), and Russia (Bogomazova et al., 2019). Furthermore, observations are indicating a long persistence of *S*. Infantis in broiler farms and increased resistance against cleaning and disinfection procedures (Asai et al., 2007; Nógrády et al., 2007, 2008; Ross and Heuzenroeder, 2008; Pate et al., 2019). Thus, we were interested whether recent *S*. Infantis organisms gained new properties resulting in the modified characteristics.

There is evidence that the acquisition of a conjugative mega-plasmid provides the bacteria with new resistance properties (EFSA, 2020) but might also confer virulence-associated characteristics, higher resistance against heavy metals or disinfectants and fitness characteristics (Aviv et al., 2014).

In view of the increased prevalence of *S*. Infantis also in German broiler production in recent years (EFSA, 2020), the question raised on possible reasons. Therefore, this study aimed to characterize and compare *S*. Infantis strains originated from different broiler farms in Germany from 20-years distant decades, the 1990s and the 2010s. *S*. Infantis organisms isolated in different decades were phenotypically characterized by serotyping and determining the antimicrobial susceptibility. In this study, whole genome sequencing (WGS) and bioinformatics analysis were used to describe the genetic traits of *S*. Infantis strains from different German broiler farms collected from 20-years distant decades.

## Materials and Methods

### Strain Selection

In this study, we analyzed a dataset consisting of 30 *S*. Infantis strains that cover a wide range of broiler farms in Germany (Table 1). Eighteen isolates were collected during the 2010s (time frame: 2014–2020) and 12 strains were isolated two decades earlier, in the 1990s (time frame: 1992–1998). *S*. Infantis strains were provided by the National Reference Laboratory for *Salmonella* at the German Federal Institute for Risk Assessment (BfR) or were received after request from regional diagnostic laboratories in different federal states in Germany. To compare the sequenced German *S*. Infantis strains with previously reported emergent *S*. Infantis clones, we searched for recent publications regarding *S*. Infantis. The criteria of selection of strains were source (broiler), region (central Europe) and, period of time when they were collected (between the 1990s and the 2010s). Thus, we downloaded sequence data of *S.* Infantis (*n* = 17) from Hungary (Olasz et al., 2015; Wilk et al., 2016, 2017) and Italy (Franco et al., 2015) (Supplementary Table S1). Beyond this criteria, we included strains from Israel where pESI was first studied (Aviv et al., 2014) and kept the strains 1326/28 (LN649235) from the United Kingdom and the non-broiler strain 335-3 (ATHK00000000) as representatives of the historical, or so-called “pre-emergent” strains. For comparison purposes, we included the recently published complete genome sequence of the *S*. Infantis strain 119944 harboring the pESI like megaplasmid from Israel (Cohen et al., 2020).

**TABLE 1:** Epidemiological data and phenotypic AMR profile of *S.* Infantis strains used in this study.

Strain	Sample	Region of isolation	Year of isolation	Phenotypic AMR profile
19PM0346	2945	Bavaria farm 1	1992	SMX
19PM0348	2947	Bavaria farm 2	1992	SMX
19PM0349	2948	Bavaria farm 3	1992	SMX
19PM0350	2949	Lower Saxony farm 1	1992	SMX
19PM0351	2951	Lower Saxony farm 2	1994	SMX
20PM0240	3222	Mecklenburg-Western Pomerania	1994	SMX
20PM0243	3225	State of Hesse farm 2	1995	SMX
20PM0245	3227	Thuringia	1995	SMX
20PM0248	3230	State of Hesse farm 3	1996	SMX
20PM0252	3234	Baden-Wuerttemberg farm 2	1997	SMX
20PM0257	3239	Rhineland-Palatinate farm 2	1998	SMX
20PM0260	3242	Lower Saxony farm 4	1998	SMX
20PM0261	3243	Bavaria farm 4	2014	SMX-CIP-TET-NAL-TGC
20PM0263	3245	Lower Saxony farm 5	2014	SMX
20PM0267	3249	Saxony-Anhalt farm 1	2015	SMX-CIP-TET-NAL-TGC
20PM0268	3250	Bavaria farm 5	2015	SMX
20PM0270	3252	Saxony-Anhalt farm 2	2015	SMX-CIP-TET-NAL-TGC
20PM0271	3253	Brandenburg farm 1	2016	SMX-CIP-TET-NAL-TGC
20PM0273	3255	Lower Saxony farm 6	2016	SMX
20PM0275	3257	Bavaria farm 6	2016	SMX-CIP-TET-NAL-TGC
19PM0355	2954	Baden-Wuerttemberg farm 1	2017	SMX-CIP-TET-NAL-TGC
19PM0358	2957	Brandenburg farm 1	2018	SMX-CIP-TET-NAL-TGC
19PM0360	2959	Bavaria farm 4	2019	SMX-CIP-TET-NAL-TGC
19PM0148	2747	Bavaria farm 5	2019	SMX-CIP-TET-NAL-TGC
19PM0149	2748	Bavaria farm 6	2019	SMX-CIP-TET-NAL-TGC
19PM0150	2749	Bavaria farm 7	2019	SMX-CIP-TET-NAL-TGC
19PM0151	2750	Baden-Wuerttemberg farm 2	2019	SMX-CIP-TET-NAL-TGC
19PM0153	2752	Baden-Wuerttemberg farm 3	2019	SMX-CIP-TET-NAL-TGC
19PM0154	2753	Bavaria farm 8	2019	SMX-CIP-TET-NAL-TGC
20PM0045	3027	Bavaria farm 6	2020	SMX-CIP-TET-NAL-TGC

*SMX, sulfamethoxazole; CIP, ciprofloxacin; TET, tetracycline; NAL, nalidixic acid; TGC, tigecycline.*

### Serotyping and Antimicrobial Susceptibility Testing

All *Salmonella* isolates were serotyped using poly- and monovalent anti-O as well as anti-H sera (SIFIN, Germany) according to the Kauffmann–White scheme (Grimont and Weill, 2007). Antimicrobial susceptibility of the *S.* Infantis strains was assessed by determining the minimum inhibitory concentration (MIC) using the broth microdilution method with Sensititre^TM^ EUVSEC plates (Trek Diagnostic Systems Ltd., East Grinstead, United Kingdom). Epidemiological cut-off values were used according to the European Committee on Antimicrobial Susceptibility Testing (EUCAST, 2018). Antimicrobial susceptibilities to sulfamethoxazole (SMX), trimethoprim (TMP), ciprofloxacin (CIP), tetracycline (TET), meropenem (MERO), azithromycin (AZI), nalidixic acid (NAL), cefotaxime (FOT), chloramphenicol (CHL), tigecycline (TGC), ceftazidime (TAZ), colistin (COL), ampicillin (AMP), and gentamicin (GEN) were examined.

### Sequencing and Bioinformatics Analysis

For paired-end sequencing with Illumina, Genomic DNA of 30 *S*. Infantis strains was extracted and purified using the QIAGEN^®^ Genomic-tip 20/G kit (QIAGEN, Germany) and the Genomic DNA Buffer Set (QIAGEN, Germany). The concentration of the DNA was determined using the Qubit dsDNA BR assay kit (Invitrogen, United States). Sequencing libraries were created using the Nextera XT DNA Library Preparation Kit (Illumina Inc., United States). Paired-end sequencing was performed on an Illumina MiSeq instrument according to the manufacturer’s instructions (Illumina Inc., United States).

For long-read sequencing with MinION, a sequencing library was prepared using the Oxford Nanopore Technologies (ONT) 1D Ligation Sequencing Kit (SQK-LSK109) with the Native Barcoding Expansion Kit (EXP-NBD104) as recommended by the manufacturer. Raw FAST5 files were processed using Guppy toolkit (v. 3.4.1) (Oxford Nanopore Technologies). The Guppy command *guppy_basecaller* was used for basecalling and *guppy_barcoder* was used for demultiplexing. *De novo* assembly for long sequencing reads was performed using Flye (v. 2.6) (Kolmogorov et al., 2019). Assembly polishing was performed with four rounds by Racon (v. 1.4.3) (Vaser et al., 2017) and one final round with Medaka (v. 0.10.0). Finally, Pilon (v. 1.23) (Walker et al., 2014) was used to correct the final assembled data from Nanopore with Illumina reads using standard settings.

To analyze the sequencing data in a standardized manner, the Linux-based bioinformatics pipeline WGSBAC was used (v. 2.0.0)^1^ (FLI_Bioinfo, 2020). Input for the pipeline was raw Illumina data and already assembled data (MinION). WGSBAC starts with quality control using FastQC (v. 0.11.7)^2^ (Andrews, 2018). Next, it calculates the raw coverage by the number of reads multiplied with their average read length and divided by the genome size. WGSBAC performs assembly using Shovill (v. 1.0.4) (Seemann, 2018) an optimizer for SPAdes assembler (Bankevich et al., 2012).

The quality of the assembled genomes is then checked using QUAST (v. 5.0.2) (Gurevich et al., 2013). Genome annotation is made by Prokka (v. 1.14.5). In order to identify contamination, the pipeline uses Kraken 2 (v. 1.1) (Wood et al., 2019) and the database Kraken2DB to classify both reads and assemblies. For *in silico* serotyping, WGSBAC utilizes SISTR (v. 1.0.2) (Yoshida et al., 2016) on the assembled genomes.

For genotyping, WGSBAC uses classical multilocus sequence typing (MLST) on assembled genomes using mlst software (v. 2.16.1)^3^ (Seemann, 2014a) that incorporates the PubMLST database^4^ (Jolley et al., 2018). Furthermore, the pipeline includes mapping based SNP-typing using Snippy (v. 4.3.6)^5^ (Seemann, 2014b) with standard settings and FastTree (v. 2.1.10) (Price et al., 2009) to calculate phylogenetic trees from SNPs. To infer phylogeny based on core genome multilocus sequence typing (cgMLST), we used the external software Ridom Seqsphere+ (v. 5.1.0) (Junemann et al., 2013) with default settings and the specific core genome scheme (cgMLST v2) for *Salmonella enterica* with 3,002 target loci developed by Enterobase (Alikhan et al., 2018).

For detection of antimicrobial resistance genes (AMR), virulence factors and plasmid replicon genes, WGSBAC uses Abricate (v. 0.8.10)^6^ (Seemann, 2015) and the databases: ResFinder (Zankari et al., 2012) and NCBI (Feldgarden et al., 2019a), Virulence Factor Database (VFDB) (Chen et al., 2005) and PlasmidFinder (Carattoli et al., 2014), respectively. For the detection of point mutations in the gene *gyrA* leading to AMR, we used the software AMRFinderPlus (v. 3.6.10) (Feldgarden et al., 2019b).

For a deeper molecular characterization of the strains, we downloaded specific pESI119944-encoded gene sequences and created customized databases for Abricate (Supplementary Table S2). These databases include sequences of genes encoding for *Salmonella* pathogenicity islands (SPIs), Ipf and K88-like fimbrial clusters and pESI fitness determinants as the toxin-antitoxin (T/AT) system (CcdAB and PemK/MazF) and the mercury operon. Furthermore, for plasmid typing, allele sequences for incompatibility groups of plasmids IncI1 (five loci) and IncF RST (seven loci) were downloaded from the Plasmid PubMLST database^7^ (Carattoli and Hasman, 2020). In order to complete the plasmid genomic characterization and to test the chimeric nature of pESI-like plasmids as described before (Aviv et al., 2014), we tested the detection of the gene sequence encoded for RepFIB replication protein A (*rep*B) and the oriV of IncP1 plasmids. We used the external software Geneious Prime (v. 2019.2.3)^8^ to complete the plasmid strain annotation and for visualization of its main genomic features.

Two phylogenetic trees were constructed for the pESI-like positive strains using Snippy to study the plasmid SNP-based phylogeny. One tree using the plasmid sequence (CP047882) as reference and a second one using the chromosome sequence (CP047881) as reference of the complete genome sequence of SI119944 strain (Cohen et al., 2020). Trees were compared using the tanglegram function of the tool Dendroscope (v 3.5.9) (Huson and Scornavacca, 2012).

## Results

### Serotyping and Antimicrobial Susceptibility Testing

All isolates were typed according to the Kauffmann–White scheme and revealed the complete antigenic formula (6, 7: r: 1, 5) for *S.* Infantis. As listed in Table 1, all *S*. Infantis strains from the 1990s were only resistant against SMX. Among the isolates from the 2010s, three strains were resistant to SMX and 15 were multidrug-resistant to SMX, CIP, TET, NAL, and TGC.

### Genomic Features of Genomes of *S*. Infantis Strains

WGS of the 30 *S.* Infantis strains revealed general genomic characteristics and allowed genoserotyping of the strains (Supplementary Table S3). We sequenced an average of 1,439,617 reads per sample (range: 524,688–2,509,738). On average, assembled genomes consisted of 54 contigs (range: 37–91) with an average read-coverage of 68 fold (range: 24–128). The average genome size was 4.8 Mbp (range: 4.6–4.9 Mbp), GC content was 52.2% and N50 values average 257,721 (range: 90,139–445,475). Kraken2 on Illumina reads classified an average of 95.31% of reads as “*Salmonella*” on the genus level and an average of 94.42% of reads as “*Salmonella enterica*” at the species level. To confirm the serological serotyping, SISTR was used for *in silico* molecular typing and predicted serovar Infantis for all the strains included in the study corroborating the phenotypic findings.

#### Genotyping and Phylogeny of *S*. Infantis Strains

**FIGURE 1:**
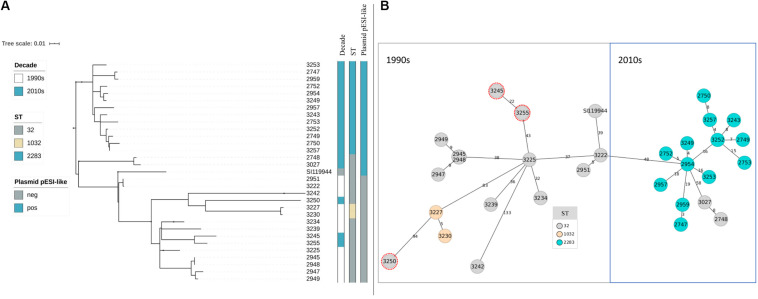
**(A,B)** Phylogeny of broiler-derived *S*. Infantis strains in Germany and *S*. Infantis SI119944 from Israel. **(A)** Phylogenetic tree based on SNPs representing two main decade- and ST-related groups as the minimum spanning tree based on cgMLST. **(B)** Plasmid pESI-like is presented in the majority of samples from the 2010s (*n* = 15) and absent for the data from the 1990s. Samples 3227 and 3230 that belong to ST1032 are grouped within the 1990s group as they do not present the plasmid pESI-like. Note that samples 2748 and 3027 belong to ST32 but they are grouped with the samples collected in the 2010s **(B)** and that three strains from the 2010s are included within the group of the 1990s as they have ST32 (marked in red in **B**).

After assessing the general sequencing characteristics, classical MLST on the assembled genomes was carried out to get a broad overview of the *S*. Infantis genotypes (Table 2). Among the complete dataset, 15 out of 30 isolates examined belong to ST32. The remaining 15 belong to two single-locus variants of ST32, namely ST2283 (in the gene *sucA*) and ST1032 (in the gene *dnaN*). The majority of the strains from the 1990s belong to ST32 (*n* = 10) while two strains belong to ST1032. The majority of the strains from the 2010s belong to ST2283 (*n* = 13) while five strains belong to ST32. Within the dataset from the 2010s, only two strains that belong to ST32 revealed the same MDR pattern as *S*. Infantis strains with ST2283. For a deeper phylogenetic analysis of the 30 *S*. Infantis strains, a minimum spanning tree based on core genome MLST (cgMLST) and a phylogenetic tree based on single nucleotide polymorphisms (SNPs) were constructed. Although both approaches differ strongly, they produced similar results (Figures 1A,B). Both phylogenetic approaches group the strains into two distinct decade- and ST-related groups except for samples 2748 and 3027 that belong to ST32 but they are grouped with the samples collected in the 2010s (Figure 1B) and three strains from the 2010s that are included within the group of the 1990s as they have ST32 (marked in red in Figure 1B). On average, the distance between the strains was 145 SNPs and 76 alleles.

**TABLE 2:** Sequence type (ST), resistance genes (ESIr pattern and other) and plasmid replicons detected in broiler-derived *S*. Infantis strains from Germany and comparison with the complete genome sequence of plasmid pESI detected in *S*. Infantis SI119944 strain from Israel.

Sample	Year of isolation	MLST (ST)	Resistance genes pattern (ESIr)	Other resistance genes	Plasmid replicon
2945	1992	32	−	*aac(6′)-Iaa*	*–*
2947	1992	32	−	*aac(3)VIa*, *ant(3“)-Ia*, *sul1, aac(6′)-Iaa*	IncI1-I(Gamma)
2948	1992	32	−	*aac(6′)-Iaa*	*–*
2949	1992	32	−	*aac(3)VIa*, *ant(3“)-Ia*, *sul1, aac(6′)-Iaa*	IncI1-I(Gamma), IncFIC(FII), IncFII(pSFO)
2951	1994	32	−	*aac(6′)-Iaa*	*–*
3222	1994	32	−	*aac(6′)-Iaa*	*–*
3225	1995	32	−	*aac(6′)-Iaa*	*–*
3227	1995	1032	−	*aac(6′)-Iaa*	*–*
3230	1996	1032	−	*aac(6′)-Iaa*	*–*
3234	1997	32	−	*aac(6′)-Iaa*	*–*
3239	1998	32	−	*aac(6′)-Iaa*	*–*
3242	1998	32	−	*aac(6′)-Iaa*	*–*
3243	2014	2283	+	*aac(6′)-Iaa, gyrA-*S83Y	IncFIB(pN55391)
3245	2014	32	−	*aac(6′)-Iaa*	*–*
3249	2015	2283	+	*aac(6′)-Iaa, gyrA-*S83Y	IncFIB(pN55391)
3250	2015	32	−	*aac(6′)-Iaa*	*–*
3252	2015	2283	+	*aac(6′)-Iaa, gyrA-*S83Y	IncFIB(pN55391)
3253	2016	2283	+	*aac(6′)-Iaa, gyrA-*S83Y	IncFIB(pN55391)
3255	2016	32	−	*aac(6′)-Iaa*	*–*
3257	2016	2283	+	*aac(6′)-Iaa, gyrA-*S83Y	IncFIB(pN55391)
2954	2017	2283	+	*aac(6′)-Iaa, gyrA-*S83Y	IncFIB(pN55391)
2957	2018	2283	+	*aac(6′)-Iaa, gyrA-*S83Y	IncFIB(pN55391)
2959	2019	2283	+	*aac(6′)-Iaa, gyrA-*S83Y	IncFIB(pN55391)
2747	2019	2283	+	*aac(6′)-Iaa, gyrA-*S83Y	IncFIB(pN55391)
2748	2019	32	+	*aac(6′)-Iaa, gyrA-*S83Y	IncFIB(pN55391)
2749	2019	2283	+	*aac(6′)-Iaa, gyrA-*S83Y	IncFIB(pN55391)
2750	2019	2283	+	*aac(6′)-Iaa, gyrA-*S83Y	IncFIB(pN55391)
2752	2019	2283	+	*aac(6′)-Iaa, gyrA-*S83Y	IncFIB(pN55391)
2753	2019	2283	+	*aac(6′)-Iaa, gyrA-*S83Y	IncFIB(pN55391)
3027	2020	32	+	*aac(6′)-Iaa, gyrA-*S83Y	IncFIB(pN55391)
**SI119944**	**2008**	**32**	**+**	***aac(6′)-Iaa, dfrA14, gyrA-*D87Y**	**IncFIB(pN55391)**

#### Resistance and Virulence Genes Patterns

The analysis of the genome sequences revealed an AMR gene pattern specific for the 15 MDR strains from the 2010s. We named this pattern ESIr (Emergent *S*. Infantis resistance pattern) and it consists of the AMR genes: *ant(3“)-Ia* (aminoglycoside resistance), *sul1* (sulfonamide resistance), *tet(A)* (TET resistance) and *qacEdelta1* [quaternary ammonium compound (QAC) and disinfectant resistance] (Table 2). Additionally, we detected a point mutation in the gene *gyrA* (*gyrA*-S83Y) leading to resistance against (fluoro)quinolones (Supplementary Table S4). The remaining three strains from the 2010s dataset did not reveal any of these resistance genes. Two strains from the 1990s, (2947 and 2949) carry the genes *ant(3“)-Ia*, *sul1* and *qacEdelta1* as well as the gene *aac(3)-VIa* (aminoglycoside resistance) but not the gene *tet(A)*. The chromosomally encoded gene *aac(6’)-Iaa* was detected among all the samples included in this study.

We detected a specific gene pattern of virulence genes among the 15 MDR strains from the 2010s (Table 3). We named this pattern ESIv (Emergent *S*. Infantis virulence) which consists of genes associated with the fimbrial clusters: K88-like fimbria (Klf) and the *S*. Infantis plasmid-encoded fimbria (Ipf) (Aviv et al., 2017). Moreover, the pattern includes genes encoding for the virulent yersiniabactin operon (*fyu*A, *irp*1, *irp*2, *ybt*A, *ybt*E, *ybt*P, *ybt*Q, *ybt*S, *ybt*T, *ybt*U, *ybt*X) and the mercury (*mer)* operon (*mer*R, *mer*T, *mer*P, *mer*C, *mer*A, *mer*D, *mer*E) conferring mercury resistance. Finally, ESIv pattern consists of the gene complex *ccd*A/B and the *pem*K/I family (T/AT system). Isolates from the 1990s did not show any of these genes. Apart from the ESIv pattern, we found among all the samples from the study the presence of ten SPIs: SPIs-1-6, SPI-9, SPIs-11-12, and CS-54 (Supplementary Table S5).

**TABLE 3:** Virulence and fitness genes detected in broiler-derived *S*. Infantis strains from Germany (ESIv pattern) and comparison with the complete genome sequence of plasmid pESI detected in *S.* Infantis SI119944 strain from Israel.

Sample	Year of isolation	K88-like fimbria (Klf)	Infantis plasmid encoded fimbria (Ipf)	*mer* operon	Yersiniabactin system	Toxin/antitoxin system (T/AT)
2945	1992	−	−	−	−	−
2947	1992	−	−	−	−	−
2948	1992	−	−	−	−	−
2949	1992	−	−	−	−	−
2951	1994	−	−	−	−	−
3222	1994	−	−	−	−	−
3225	1995	−	−	−	−	−
3227	1995	−	−	−	−	−
3230	1996	−	−	−	−	−
3234	1997	−	−	−	−	−
3239	1998	−	−	−	−	−
3242	1998	−	−	−	−	−
3243	2014	+	+	+	+	+
3245	2014	−	−	−	−	−
3249	2015	+	+	+	+	+
3250	2015	−	−	−	−	−
3252	2015	+	+	+	+	+
3253	2016	+	+	+	+	+
3255	2016	−	−	+	−	−
3257	2016	+	+	+	+	+
2954	2017	+	+	+	+	+
2957	2018	+	+	+	+	+
2959	2019	+	+	+	+	+
2747	2019	+	+	+	+	+
2748	2019	+	+	+	+	+
2749	2019	+	+	+	+	+
2750	2019	+	+	+	+	+
2752	2019	+	+	+	+	+
2753	2019	+	+	+	+	+
3027	2020	+	+	+	+	+
**SI119944**	**2008**	**+**	**+**	**+**	**+**	**+**

As previously reported (Bogomazova et al., 2019), similar resistance and virulence gene patterns have been determined by the presence of a pESI-like plasmid carried by emergent *S*. Infantis strains. Likely, in the case of the strains of this study, the presence of a pESI-like plasmid could explain this genetic profile. Therefore, we aimed at the genomic detection and further characterization of this pESI-like plasmid among our strains.

#### Detection, Genomic Characterization and Phylogeny of a pESI-Like Plasmid

First, we scanned for replicon sequences of plasmids (Supplementary Table S6). All the 15 MDR strains positive to ESIr and ESIv patterns, presented the replicon IncFIB (pN55391) (Table 2). A genomic in-depth analysis for the typification of the plasmids revealed that the 15 strains positive for the IncFIB(pN55391) replicon, had the profile: *ardA*2, *pilL*3, *sogS*9, *trbA*21 while *repI*1 was absent. However, they were positive for the RepFIB replication protein A (*rep*B). Besides, they were positive for the detection of the sequence of the plasmid RK2 (from *E. coli*) DNA transposon (Tn1723) insertion sites (M20134) revealing the chimeric nature of the pESI-like plasmid as described previously (Aviv et al., 2014, 2016).

The samples 2947 and 2949 from the 1990s were positive for replicon IncI1-I(Gamma) and had the complete IncI gene profile: *ardA*4, *pilL*1, *sogS*2, *trbA*13, *repI*1. Sample 2949 simultaneously carries IncFIC(FII) and IncFII(pSFO) replicons and two alleles of IncF RST: FII91 and FIC3 (Supplementary Table S6). Interestingly, sample 3255 from the 2010s, was negative for the presence of IncFIB (pN55391); however, it carried additionally three different replicons: IncFIC(FII), IncFII(S) and IncFII(SARC14) for IncF plasmids (Supplementary Table S6). Sample 3255 did not present any gene from the IncI1 scheme, nor *rep*B, but two genes from the IncF RST scheme: FIIS5 and FIC3. The remaining non-resistants trains from the dataset did not contain any gene for an incompatibility group of plasmids.

**FIGURE 2:**
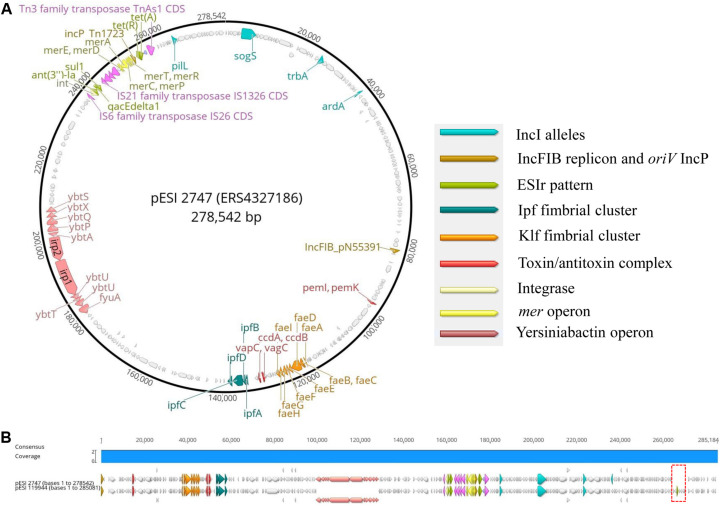
**(A,B)** Main genomic traits and alignment of the complete plasmid sequence of pESI119944 from the Israelian strain SI119944 and pESI2747 from the German strain 19PM0148. The red frame indicates the non-common fragment in the alignment.

Second, to add further evidence that the 2010s prevalence of *S*. Infantis in German broilers may be due to the presence of a pESI-like megaplasmid, we downloaded and examined the complete genome sequence of the megaplasmid pESI119944 found for the first time in the strain SI119944 in Israel (Aviv et al., 2014). Indeed, the analysis of the complete sequence of this plasmid showed the presence of the replicon IncFIB(pN553391), the allele profile of an IncI, the origin of replication of an IncP and *rep*B (Supplementary Table S6). As shown in Tables 2, 3, main resistance and virulence traits detected among the German strains positive for replicon IncFIB(pN553391), were also present in the Israelian strain SI119944. Third, to have an in-depth comparison of the pESI-plasmid found within the German strains, we performed Oxford Nanopore sequencing of the sample 2747 that represent the samples from the 2010s and meets the characteristics described above regarding resistance, virulence genes, and plasmid nature. The hybrid assembly of sample 2747 resulted in two closed contigs corresponding to the chromosome (4,678,881 bp length) and the pESI-like plasmid (278,542 bp length) that we designated as pESI2747. The N50 value of the genome was 4,678,881 bp and the GC content was 52.18%. Kraken2 gave a match of 100% for *Salmonella enterica*. Figures 2A,B show the main features of the alignment of pESI119944 from Israel and pESI2747 from Germany. The alignment shows a consensus sequence of ∼285,184 bp. This consensus sequence consists of a common fragment of ∼277,693 bp (∼97.37%) between both sequences and a non-common region of ∼7,301 bp (∼2.56%). The *repB* gene coding for RepFIB replication protein A is located in the positions 80,555 and 81,580 bps in pESI2747, while on pESI119944 it is located at the beginning of the plasmid. Moreover, the genes *ardA*, *pilL*, *sogS*, *trbA* were found as well in different locations along pESI2747 compared to pESI119944. The sequence of an origin of replication for IncP plasmid was found as well in both plasmid sequences between the *mer* operon and the resistance genes *tet*(A) and *tet*(R) as previously described (Aviv et al., 2014, 2016). In pESI2747, we found the ESIr and the ESIv pattern common for the 15 MDR strains from the 2010s. Resistance genes *ant(3“)-la*, *sul1* and *qacEdelta1* were located together in a region flanked by an IS6 transposase IS26, Integrase/recombinase (int) (Uniprot: P62592)^9^ and IS21 family transposase IS1326. Resistance genes to TET *tet(A*) and *tet(R)* were found as well together, having upstream the Tn3 family transposase TnAs1. In the non-common part, we found the TMP resistance encoding gene *dfrA14* only presented in the sequence of pESI119944. Regarding virulence, we found the k88-like fimbria (Klf) cluster on the sequences of both plasmids spanning a region of ∼8,000 bp and the Ipf cluster that occupies a region of 5,100 bp. Between them, we found the gene cluster *ccd*A-*ccd*B encoding for the toxin/antitoxin system and the *vagC* and *vapC* genes. The *pemK-pemI* is located in position 95,086. Moreover, we found the 11 genes of the yersiniabactin operon spanning a region of ∼29,000 bp.

To study, if all German strains potentially carrying pESI have a similar plasmid structure as pESI2747, we mapped the Illumina short reads to the sequence of pESI119944 and analyzed their coverage vector (Supplementary Figure S1). We found that the positive strains cover practically the complete sequence of pESI119944 except for a gap at the end of the reference sequence suppose to be the non-common part observed between our pESI-like plasmid and the pESI119944. In summary, we add evidence that multidrug-resistant strains in the 2010s carry a pESI-like megaplasmid.

Finally, we were interested if the plasmid evolves independently or if there has occurred a co-evolution together with the chromosomes. Therefore, we studied the plasmid-based SNP phylogeny as previously performed (Alba et al., 2020) for the 15 *S.* Infantis strains harboring the pESI-like plasmid (Supplementary Figure S2). In general the plasmid phylogeny seems to be similar to the chromosomal phylogeny.

### Genomic Characteristics of *S*. Infantis Strains From Israel, Hungary, and Italy

To compare our findings with recently published results from the emergent *S*. Infantis clones, we downloaded a total of 17 genomes from studies performed in Italy (Franco et al., 2015), Hungary (Olasz et al., 2015; Wilk et al., 2016, 2017) and Israel (Aviv et al., 2014) as described above and analyzed them using our pipeline (Supplementary Table S1). Genotyping revealed that ST32 was the only sequence type of *S.* Infantis strains presented within the data from Israel, Hungary and Italy while ST2283 was not detected. PlasmidFinder found the replicon IncFIB(pN55391) in 13 out of 17 of the strains and the majority of them (*n* = 12) presented the IncI profile: *ardA*2, *pilL*3, *sogS*9, *trbA*21, and *repI*1 absent (Supplementary Table S6). Additionally, we detected two replicons (IncX1 and IncX3) in two Hungarian strains (SI240/16 and SI3337/12) and IncI1-I(Gamma) in one Italian strain (ERS846145) with IncI profile including the *repI*1. As expected, samples from Hungary SI69/04, Israel 335-3 and United Kingdom 1326/28 collected before the 2000s did contain neither plasmid replicons, nor IncI genes. Abricate using ResFinder, revealed a variety of 15 different resistance genes among the *S*. Infantis harboring the pESI-like plasmid (Supplementary Table S4). The ESIr [*ant(3“)-la*, *sul1*, *tet(A)* and *qacEdelta1*] was found among 10 out of 17 strains from the three countries. The remaining strains had a variation of this pattern and presented other additional genes. Especially, the Italian strains present a wide variety of resistance genes including resistance genes related to extended-spectrum β-lactamase (ESBL), like *blaCTX-M-1* as reported previously (Franco et al., 2015). Three out of seven Hungarian strains presented ESBL genes as well like the *blaCTX-M-14* or *blaTEM-104*.

Chromosomal mutations of gene *gyr*A were found as well: *gyrA*-S83Y (8 out of 17) and *gyrA*-D87G (4 out of 17), *gyrA*_D87Y was found only in SI119944. The ESIv pattern described above within the German *S.* Infantis was found in 12 out of 16 strains (Supplementary Table S5). Therefore, the results from this study are in line with the findings in other European countries regarding the emergence of multidrug and virulent *S*. Infantis clones.

To see if there is a clonal transmission of the strains, a minimum spanning tree based on cgMLST was constructed (Supplementary Figure S3). German *S*. Infantis strains of ST2283 form 2010s are close to two Hungarian strains that were reported as new *S*. Infantis clones (Nógrády et al., 2012). The smallest difference between external strains and German pESI-like strains is 37 alleles between the Hungarian SI54/04 and the German 2954, therefore we could not detect any clonal relatedness. The two ST32 strains from the 2010s are most closely related to two emergent *S*. Infantis strains from Italy. We could detect clonal transmission between two Italian strains where the smallest number of different alleles was 1.

## Discussion

Studies performed within and outside Europe revealed an emergent dissemination of *S*. Infantis clones in humans and several animal species (Nógrády et al., 2007, 2008, 2012; Aviv et al., 2014; Franco et al., 2015; Olasz et al., 2015; Yokoyama et al., 2015; Wilk et al., 2016; Hindermann et al., 2017; Szmolka et al., 2018; Acar et al., 2019; Bogomazova et al., 2019). There is evidence that the acquisition of a conjugative megaplasmid provides the bacteria with new resistance properties which might have contributed to the increased occurrence of this serovar. An in-depth analysis of the genetic characteristics of the plasmid called pESI (Aviv et al., 2014) or similar pESI-like plasmids (Franco et al., 2015; Szmolka et al., 2018) revealed that they also encode for virulence-associated characteristics, resistance to heavy metals or disinfectants and fitness characteristics. The analysis and genomic comparison of the complete genome sequence of SI119944 from Israel (Cohen et al., 2020) and strain 2747 from Germany demonstrate and confirm the characteristic resistance, virulent as well as fitness traits encoded on a pESI-like plasmid. Furthermore, results from this study confirm the observed switch in the occurrence of *S*. Infantis organisms in broilers from non-MDR strains screened until the 2000s (Asai et al., 2007; Shahada et al., 2008; Wilk et al., 2017) to the emergence of MDR clones collected during and after the end of the 2000s (Nógrády et al., 2007, 2008, 2012). ST32 is a highly conserved sequence type of *S*. Infantis (Monte et al., 2019) and was the dominating MLST type isolated from various and numerous sources (broilers, pigs, cattle, food, human) in different European countries (Hauser et al., 2012; Hindermann et al., 2017; Gymoese et al., 2019). In this study, we describe the occurrence of two single locus variants of ST32 within the *S*. Infantis from German broiler production: ST2283 and ST1032. The emergence of ST2283 in *S*. Infantis organisms from the 2010s is linked with the presence of a pESI-like plasmid that confers a MDR pattern that has not been found among *S*. Infantis ST32 strains originating from the 1990s. However, we also identified two ST32 strains of *S*. Infantis from the 2010s harboring the resistant coding plasmid. We observed general concordance in cluster separation between the chromosome-based tree and plasmid-based tree in all strains harboring pESI-like megaplasmid (ST32 and ST2283) as shown also by Alba et al. (2020). Therefore, we hypothesize that the plasmid has co-evolved with the chromosome and both STs gained the plasmid in two (or more) evolutionary independent events. However, a rather rare occurrence of MDR clone ST32 compared with the higher prevalence of MDR clone ST2283 in recent years indicates an obvious greater tendency of dissemination of this clone in Germany and perhaps European broiler production, and therefore, another until now not detected property of ST2283. We also detected two non-resistant ST1032 clones from the 1990s. This variant of ST32 had been described before in a non-resistant isolate from food (Alba et al., 2020). In this study, bioinformatics analysis revealed a correlation between the resistance gene pattern named as ESIr [*ant(3“)-la, sul1, tet(A*)] and the phenotypic resistance profile to aminoglycosides, sulfonamides, and TETs. Furthermore, gen *aac(6’)-Iaa* was not located on pESI119944 but on the chromosome. In line with the literature, we found the gene *aac(6’)-Iaa* to be chromosomal-encoded gene (Salipante and Hall, 2003). On the other hand, German broiler derived *S.* Infantis strains showed phenotypic resistance to quinolones like CIP and NAL. Genotype findings do correlate with the phenotypic results as we detected the well-studied mutation in *gyrA* gene that codifies for resistance to those antibiotics (Chen et al., 2019). The predominant MDR pattern found among the emergent *S*. Infantis clones from Europe consists mainly of antimicrobials that belong to the major classes of antibiotics FOT, CIP, cephalosporin, TET, sulfonamide, fluoroquinolone, and TMP (Cloeckaert et al., 2007; Franco et al., 2015; Acar et al., 2019). Phenotypic and genotypic variants of this pattern have been observed in Hungary related to two different pulsotype clusters (Nógrády et al., 2012). Variants of this MDR pattern including ESBL resistant isolates of *S*. Infantis were also found in Italy and Germany (Franco et al., 2015; Fischer et al., 2017). Resistance and virulence traits coevolved and interfere in the ecology of a strain (Beceiro et al., 2013). Thus, the increasing emergence of a strain is not only dependent on antimicrobial resistance, but also on virulence, bacteriocin secretion, biocide resistance and, biofilm formation (Acar et al., 2019). Consequently, in this study, the bioinformatics analysis for virulence determinants showed a common pattern in virulence and fitness genes within the MDR isolates. *Salmonella* pathogenicity islands and other different gene complexes that encode for fimbriae production, adherent and non-adherent products, as well as curli structures, are of special interest because of their involvement in host colonization, persistence, motility, and invasion (Barnhart and Chapman, 2006; Rychlik et al., 2009; Aviv et al., 2017). The strong dissemination of *S*. Infantis not only in broilers during the last two decades wonders whether the increased antimicrobial resistance, the swift in MLST type, the virulence properties, the capability of biofilm formation or other unknown factors are responsible for this emergence. Different hypotheses try to explain this phenomenon. For example, it is suspected that the increased prevalence of *S.* Infantis could be due to the general decreased prevalence of *S*. Enteritidis in poultry farms (Szmolka et al., 2018). It is also suggested that the EU trade of broiler chicken and the pyramidal structure of the poultry industry may be factors of the rapid spread of emergent clones of *S.* Infantis carrying the pESI-like plasmid beyond national borders (Alba et al., 2020; Nagy et al., 2020). The long term use of special groups of antimicrobial substances might have resulted in selection pressure and increased emergence of particular bacterial organisms (Nógrády et al., 2007, 2012). However, it is also stated that antimicrobial usage is not always linked to a higher *Salmonella* prevalence (Asai et al., 2007). The acquisition of the megaplasmid pESI does not result in a significant burden to its hosts as it is presented only as a single copy in the bacteria genome, therefore, it does not seem to limit the dissemination of the organisms (Aviv et al., 2016).

Production of fimbriae and the ability to form biofilms are discussed as factors enabling a long term persistence of *Salmonella* organisms at poultry farms. The gene *fyuA* (Schubert et al., 1998) together with the genes *irp1* and *irp2* are involved in biofilm formation (Hancock et al., 2008). In this study, gene *irp1* was found together with *irp2* in all strains that carry the pESI-like plasmid suggesting a possible role in persistence of *S.* Infantis organisms. However, the association of yersiniabactin and biofilm in serovar Infantis has not been yet wide studied in contrast with other microorganisms as in Uropathogenic *Escherichia coli* (UPEC) (Zamani and Salehzadeh, 2018). It has been demonstrated before a higher biofilm formation for pESI positive strains (Aviv et al., 2014). Besides, very recently, it has been demonstrated a higher cell adhesion of *S*. Infantis compared with other serovars. Resistance to heavy metals or biocides like QACs might also play a role in the emergence of *S*. Infantis. The gene *qacEdelta1* (Chuanchuen et al., 2007), located on pESI-like megaplasmids codes for resistance against QACs and was found in this study exclusively in strains from the 2010s. On the other hand, detailed analysis of the sequence of pESI-like pESI2747 has revealed not only resistance genes but also virulence genes, toxin/antitoxin systems that as previously described (Aviv et al., 2014; Acar et al., 2017) play a clue role in the emergence of *S.* Infantis in poultry. However, it is open whether the encoded resistance against disinfectants might contribute to a higher *S*. Infantis persistence at broiler farms.

In conclusion, broiler derived *S.* Infantis strains of ST2283 in Germany show similarities to emergent *S*. Infantis strains from Europe including the possession of the pESI-like megaplasmid which encodes for antimicrobial resistance, virulence genes (fimbrial clusters) and fitness determinants (toxin/antitoxin system) that enhance bacterial adaptability. Therefore, the involvement of the megaplasmid might explain the current spread of these emergent *S*. Infantis organisms. However, specific reasons for a suspected higher persistence of MLST2283 could not be identified in this study. It seems that *S*. Infantis persistence in broiler farms is caused by its occurrence in the primary broiler production and ineffective cleaning and disinfection protocols at least for these special clones. Epidemiological studies on the occurrence of *S.* Infantis ST2283 in the whole broiler production chain and the establishment of effective control measures are essential to prevent these organisms from entering the food chain.

## Data Availability Statement

The original contributions presented in the study are publicly available. Sequencing data of this study can be found at https://www.ebi.ac.uk/ena/data/view/PRJEB36784. The datasets presented in this study can be found in online repositories. The names of the repository/repositories and accession number(s) can be found in the article/Supplementary Material.

## Author Contributions

UM conceived and coordinated the study, performed serotyping and MIC determination, and wrote the manuscript. JL coordinated the study, developed and implemented the bioinformatics pipeline, and wrote the manuscript. SG-S performed the bioinformatics analysis, performed in-depth analysis of the plasmid, and wrote the manuscript. MA-G performed MinION sequencing and data analysis. HT critically read the manuscript. All authors read and approved the final manuscript.

## Conflict of Interest

The authors declare that the research was conducted in the absence of any commercial or financial relationships that could be construed as a potential conflict of interest.

## Footnotes

1 https://gitlab.com/FLI_Bioinfo/WGSBAC

2 https://www.bioinformatics.babraham.ac.uk/projects/fastqc/

3 https://github.com/tseemann/mlst

4 https://pubmlst.org/salmonella

5 https://github.com/tseemann/snippy

6 https://github.com/tseemann/abricate

7 https://pubmlst.org/plasmid/

8 https://www.geneious.com

9 https://www.uniprot.org/

## Supplementary Material

FIGURE S1 Mapping coverage of all pESI-like positive strains across the complete genome sequence of plasmid pESI119944.

FIGURE S2 Tanglegram plot representing the comparison between pESI-like positive strains using the chromosome sequence of *S*. Infantis ESI119944 (CP047881) (left) as reference and the plasmid pESI119944 sequence (CP047882) as reference (right).

FIGURE S3 Minimum spanning tree for previously reported emergent *S*. Infantis clones from Israel (Aviv et al., 2014, 2016), Hungary (Olasz et al., 2015; Wilk et al., 2016, 2017), and Italy (Franco et al., 2015) used in this study based on cgMLST.

TABLE S1 *S*. Infantis genomes for previously reported emergent *S*. Infantis clones from Israel (Aviv et al., 2014, 2016), Hungary (Olasz et al., 2015; Wilk et al., 2016, 2017), and Italy (Franco et al., 2015) used in this study and their general genome characteristics and genoserotyping.

TABLE S2 Specific gene sequences downloaded for the creation of customized databases for Abricate and available databases downloaded. These databases include sequences of genes encoding for Salmonella pathogenicity islands (SPIs), fimbrial clusters Ipf and K88-like and pESI encoded fitness determinants as the toxin-antitoxin (T/AT) system (CcdAB and PemKI/MazF), mercury operon and allele sequences for incompatibility groups plasmids IncI1 and IncF for plasmid typing.

TABLE S3 Whole Genome Sequencing general characteristics and genoserotyping of the *S*. Infantis strains from Germany used in this study.

TABLE S4 Resistance genes and chromosomal point mutations found among the *S*. Infantis strains used in this study.

TABLE S5 Virulence genes, SPIs, fimbrial cluster genes and fitness genes found among the *S*. Infantis strains used in this study.

TABLE S6 Plasmid replicons, plasmid pMLST typing and genes encoding for plasmid origin of replication found among the *S*. Infantis strains used in this study.
