# Supplementary figures and images for: Emergence of Multidrug-Resistant Salmonella enterica Subspecies enterica Serovar Infantis of Multilocus Sequence Type 2283 in German Broiler Farms
Source: Front Microbiol. 2020 Jul 17;11:1741. doi: 10.3389/fmicb.2020.01741 (PMC7380084; doi:10.3389/fmicb.2020.01741)

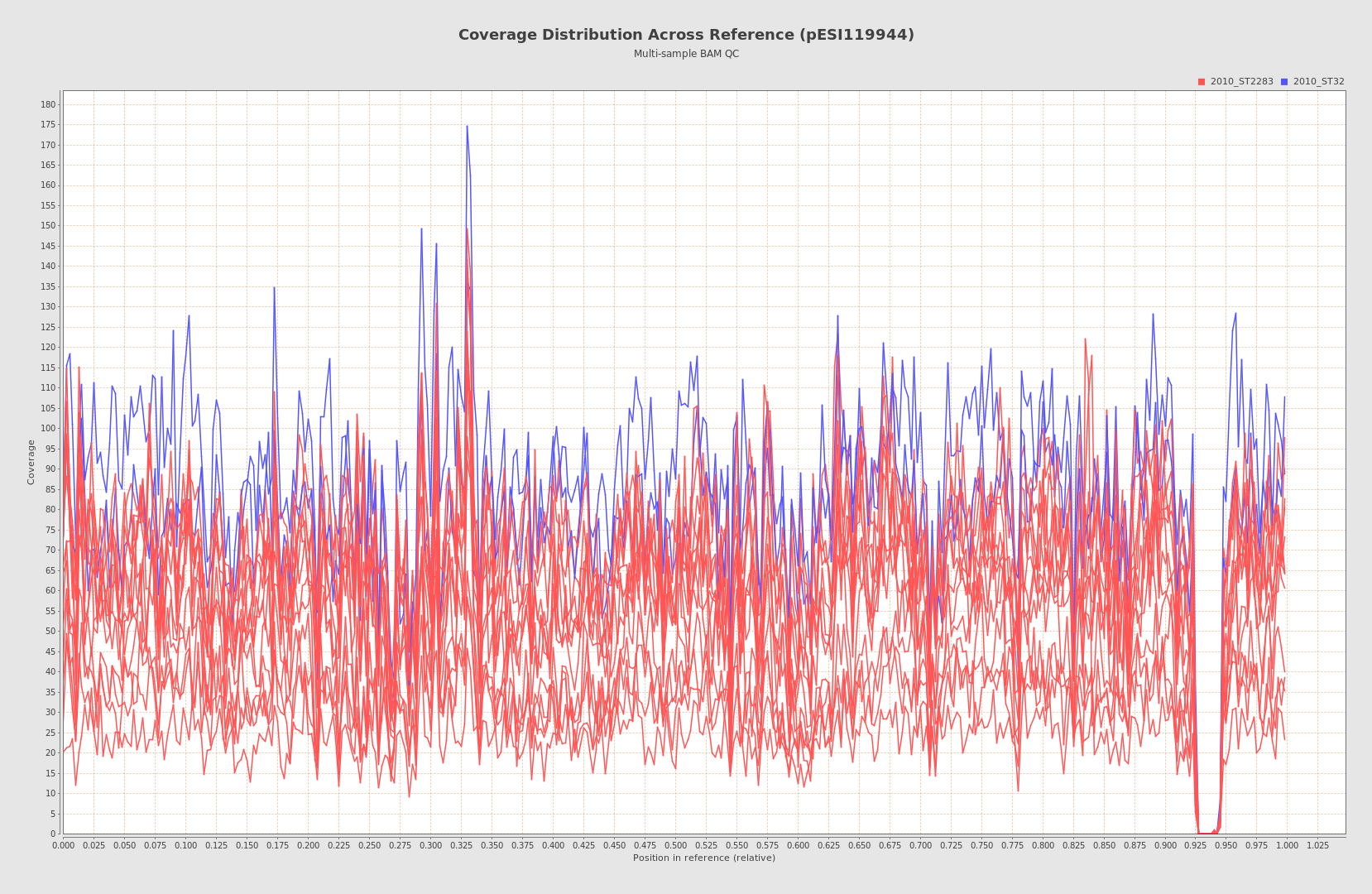

Supplement: FIGURE S1 — Mapping coverage of all pESI-like positive strains across the complete genome sequence of plasmid pESI119944. [file Data_Sheet_1.ZIP › Supplementary Material Presentation/Supplementary Figure 1.png]

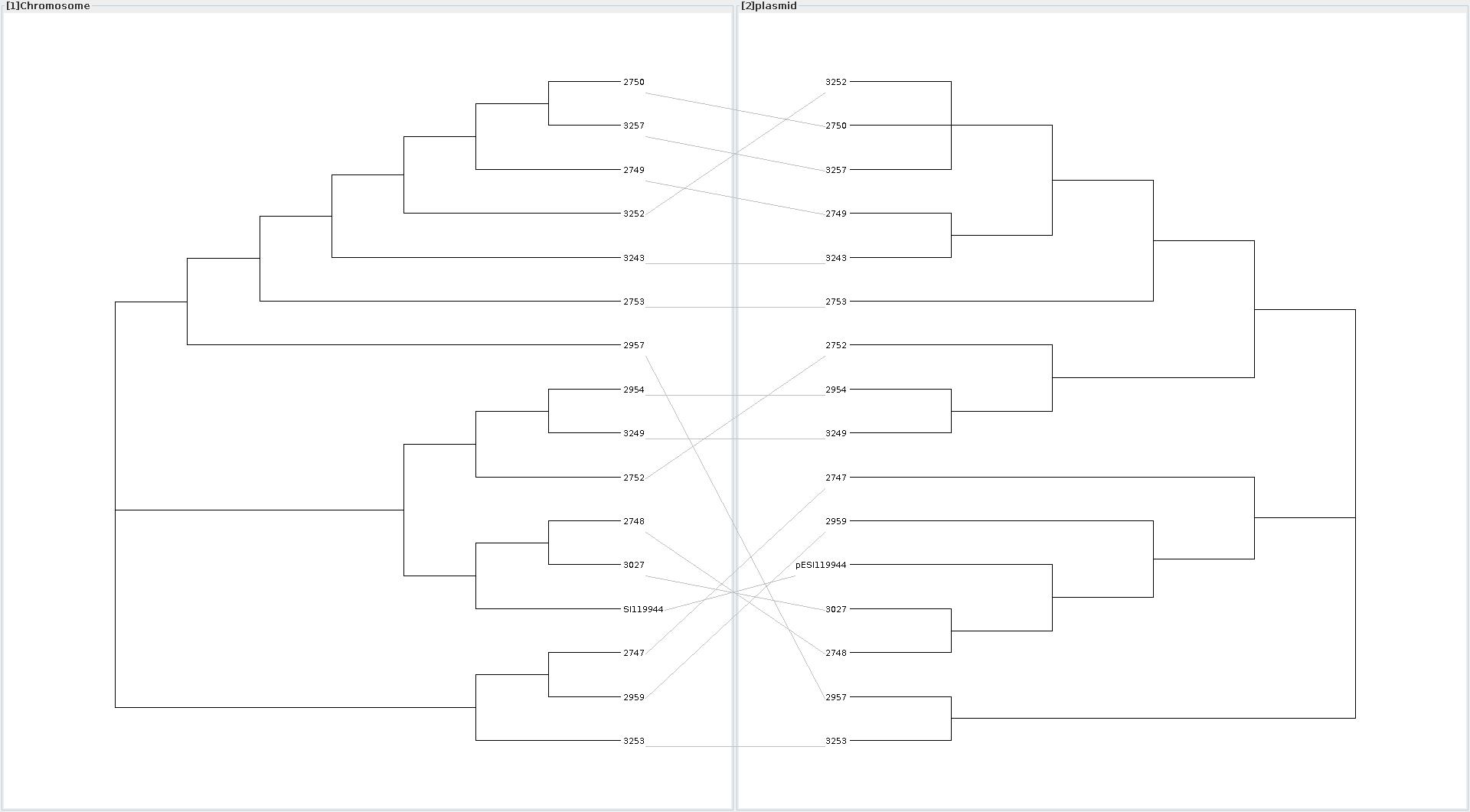

Supplement: FIGURE S1 — Mapping coverage of all pESI-like positive strains across the complete genome sequence of plasmid pESI119944. [file Data_Sheet_1.ZIP › Supplementary Material Presentation/Supplementary Figure 2.jpg]

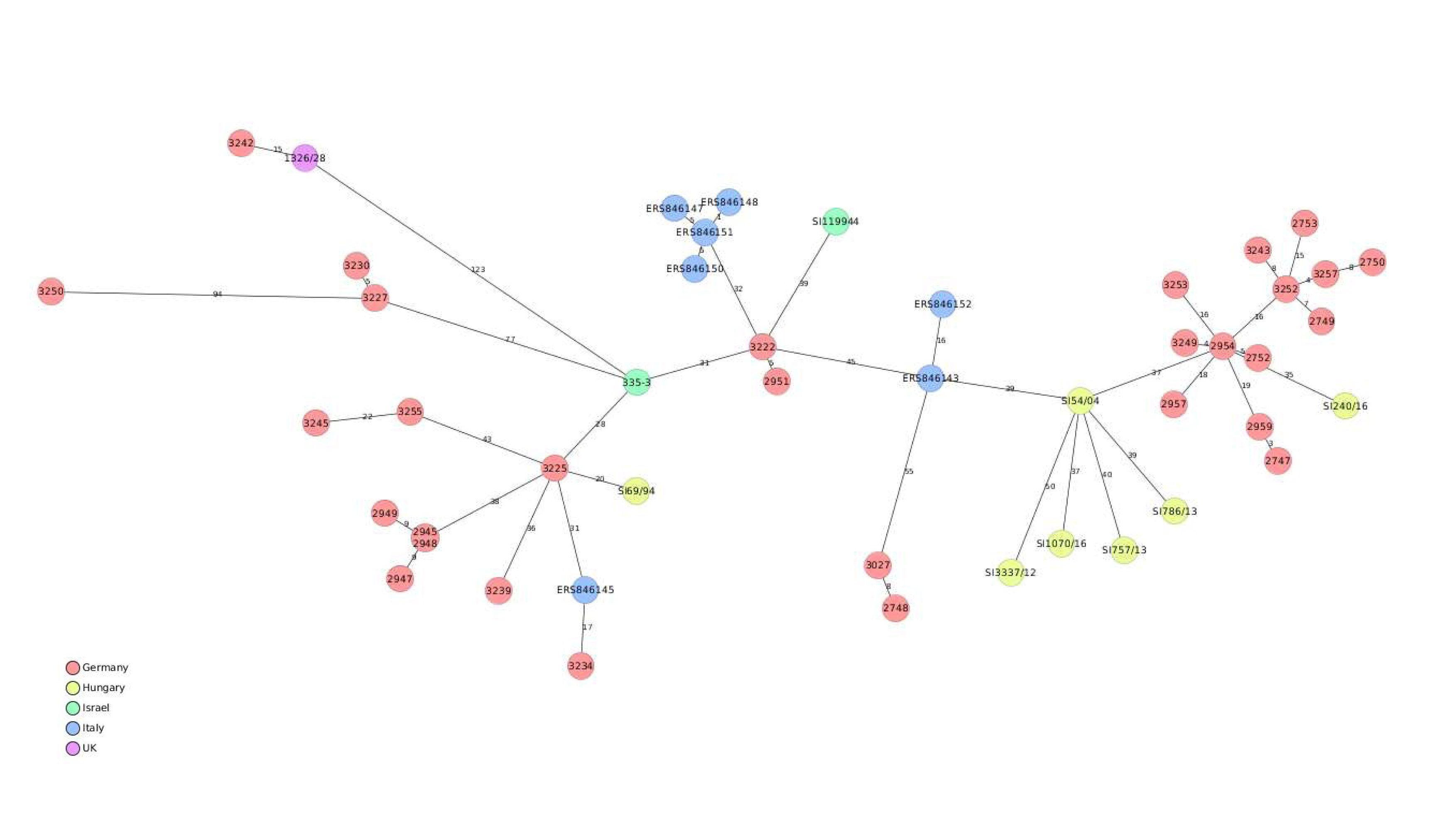

Supplement: FIGURE S1 — Mapping coverage of all pESI-like positive strains across the complete genome sequence of plasmid pESI119944. [file Data_Sheet_1.ZIP › Supplementary Material Presentation/Supplementary Figure 3.jpg]
